# Characterization of the novel In1059 harbouring VIM gene cassette

**DOI:** 10.1186/s13756-017-0204-1

**Published:** 2017-05-18

**Authors:** Dongguo Wang, Jinhong Yang, Meiyu Fang, Wei He, Ying Zhang, Caixia Liu, Dongsheng Zhou

**Affiliations:** 1 Department of Clinical Laboratory Medicine, Taizhou Municipal Hospital affiliated with Taizhou University and the Institute of Molecular Diagnostics of Taizhou University, 381 Zhongshan Eastern Road, Taizhou, Zhejiang 318000 China; 2 Department of Clinical Laboratory Medicine, the Second Affiliated Hospital and Yuying Children’s Hospital of Wenzhou Medical University, 109 Xuanyue Western Road, Wenzhou, Zhejiang 3250027 China; 3 Department of Clinical Laboratory Medicine, Hangzhou First People’s Hospital of Nanjing Medical University, 261 Huansha Road, Hangzhou, Zhejiang 310006 China; 4 Department of Clinical Laboratory Medicine, Wenzhou Hospital of Integrated Traditional Chinese and Western Medicine, 75 Jinxiu Road, Wenzhou, Zhejiang 325001 China; 5 State Key Laboratory of Pathogen and Biosecurity, Beijing Institute of Microbiology and Epidemiology, 20 Fengtai Eastern Avenue, Beijing, 100071 China

**Keywords:** Novel integron, In1059, Plasmid digestion, Cloning, Mobilization inference, Evolution

## Abstract

**Background:**

VIM-type enzyme encodes the most widely acquired metallo-β-lactamases in Gram- negative bacteria. To obtain current epidemiological data for integrons from enterobacteriae in hospital, the study characterizes the genetic structure in In1059 by comparison with In846 integrons harbouring VIM gene and other class 1 integrons including In37, In62, In843 and In1021 with the aim of identifying the putative mechanisms involved integron mobilization and infer evolution of relevant integrons.

**Methods:**

Six of 69 recombinant plasmids from clinical strains were found to be class 1 integrons by digestion with BamHI, drug susceptibility testing, conjugation experiments, PCR amplification, integron cloning and sequencing, genome comparison, and detection of carbapenemase activity.

**Results:**

The sequences of the six recombinant plasmids encoding In1021, In843, In846, In37, In62, and the novel In1059 integron had approximate lengths of ~4.8-, 4.1-, 5.1-, 5.3-, 5.3- and 6.6- kb, respectively. The genetic structures of these integrons were mapped and characterized, and the carbapenemase activities of their parental strains were assessed.

**Conclusions:**

Our results suggest that the six variable integron structures and regular variations that exist in the gene cassettes provide a putative mechanism for the integron changes. Our study has also shown that the genetic features in the integrons named above fall within a scheme involving the stepwise and parallel evolution of class 1 integron variation likely under antibiotic selection pressure in clinical settings.

**Electronic supplementary material:**

The online version of this article (doi:10.1186/s13756-017-0204-1) contains supplementary material, which is available to authorized users.

## Background

Multidrug resistance can occur by the acquisition of DNA, and the horizontal transfer of drug-resistance genes or drug-resistance gene arrays from one cell to another also occurs and this process transfers drug resistance genes via plasmids and transposons. In recent years, most of the resistance genes found on the plasmids and transposons of Gram-negative bacilli have been found to be integrated into DNA elements called integrons [1]. These integrons contain potential mobile genetic elements that feature a site-specific recombination system capable of integrating and expressing gene cassette structures in the host bacterium [2].

Integrons are the most efficient genetic elements in terms of their ability to disseminate drug-resistance genes between bacteria [3]. Depending on the sequence of the *intI* gene, integrons have been confirmed to be many classes, and only class 1, 2 and 3 integrons were first identified association with mobile genetic elements [4], intIA with chromosomal integrons and VchintIA with V. cholerae chromosomal integrons [5]. By capturing exogenous cassettes and ensuring expression of the captive genes, an integron plays important role in the horizontal dissemination of antibiotic-resistance genes [6–8]. The mechanisms of integration and excision of gene casettes are well described with integrations known to occur at *attI* × *attC* recombination sites [9, 10], and excisions requiring *attC* × *attC* recombination sites, which occur in single-stranded sequences and activate the folded bottom strand [11, 12]. Because of their linkage with transposons or being plasmid encoded, class 1 integrons can capture genetic structures, express gene cassettes, and facilitate their own mobility, but they are incapable of self-mobilization [5, 13].

Integrons usually contain three functional components: an integrase gene (*intI*), a primary recombination site (*attI*), and an outward-orientated promoter (*P*_*C*_) [14]. Cassette integrations occur mainly at the *attI* site and this ensures the expression of the captured cassettes under the control of the *P*_*C*_ promoter [7, 15]. For most class 1 integrons, the 5′-CS regions are similar and include *intI* and *attI* genes; however, differences exist in the 3′-CS sequences and the three open reading frames (ORFs) [16].

VIM-type metallo-β-lactamases were first isolated from *Pseudomonas aeruginosa* and other Gram-negative nonfermenting bacteria in Europe [17–20]. These enzymes were subsequently observed worldwide in Gram-negative nonfermentative bacteria and in Enterobacteriaceae. Class 1 integrons harbouring VIM-type gene cassettes have spread among various Gram-negative pathogens [21]. Therefore, this study aimed to characterize the genetic structures of the novel In1059 integron by comparison with the In846 integron and other structural class 1 integrons from enterobacteriaceae in terms of the VIM-type gene cassettes. From this analysis, we have also identified the described mechanism underlying integron mobilization, revealing the relations amongst structures of class 1 integrons including novel In1059 and other integrons in the study.

## Methods

### Clinical bacterial isolates and drug susceptibility testing

In total, 69 non-redundant multidrug-resistant enterobacteria strains, including non-typhoidal *Salmonella* Nsa243 and Nsa217 (harbouring In37 and In1021), *Enterobacter cloacae* Ecl175 (In843), *Klebsiella pneumoniae* Kpn761 and Kp3349 (In62 and novel In1059), and *Enterobacter aerogenes* Eae634 (In846), were recovered from hospitalized patients with clinical infections. The strains were assessed for integrons at the Taizhou Municipal Hospital of China (July 2013 to July 2015). Bacterial species was identified by 16 S rRNA gene sequencing [22]. All the above integron-harbouring isolates, plus *Escherichia coli* TOP10 (Invitrogen, USA), were used in the study. *E. coli* TOP10 was used as the host for the cloning experiments and for susceptibility testing experiments.

The minimum inhibitory concentration (MIC) values of 12 antimicrobial agents, including cephalosporins (cefazolin, ceftazidime and ceftriaxone), aminoglycosides (netilmicin, tobramycin and amikacin), carbapenems (ertapenem, meropenem and imipenem), and quinolones (norfloxacin, ofloxacin and ciprofloxacin), were determined using drug susceptibility plates and the Microscan broth dilution method (Microscan, Washington, USA). The MICs were interpreted according to the Clinical and Laboratory Standards Institute guidelines [23].

### Plasmid digestion, integron cloning and DNA sequencing

Plasmids from six integron-harbouring isolates were collected using an AxyPrep Plasmid Miniprep kit (Axygen Biosciences, Beijing, China) according to the manufacturer’s instructions and according to Wang et al., 2014 [24]. The plasmids were digested with BamHI (TaKaRa, Dalian, China), and the six integron-harbouring recombinant plasmids were electrophoretically resolved to generate genetic maps.

To characterize the recombinant plasmid-encoding integrons from the above six isolates, we digested the plasmids with BamHI and ligated the relevant fragments to the pMD19-T cloning vector (TaKaRa, Dalian, China), and then transformed the ligation mixture into *E. coli* TOP10 host bacteria. Colonies that were *aacA4*-positive were isolated and the inserts within the recombinant pMD19-T vectors were sequenced using the primers specified in Table 1 and the following PCR conditions: 3 min at 94 °C, 30 cycles of 1 min each at 94 °C, 50–59 °C and 72 °C, followed by 10 min at 72 °C. The total reaction volume was 25 μL, and the eluent volume was 10 μL. After amplification, the PCR products were separated by gel electrophoresis on a 0.6% agarose gel run at 90 V for 90 min in 0.5 × TBE buffer. Next, the plasmid DNAs or fragments of different sizes that harboured integrons were recovered, and the initial positions of the relevant genes in the recombinant plasmids were determined according to a previously established method for estimating plasmid DNA sizes [25]. After that, the integron sequences involving different genes were obtained, and the genetic structures were mapped and characterized.

**Table 1:** Primers used for PCR amplification and sequencing

Primer		Sequence (5′-3′)	Length (bp)	Reference
5′-CS	F	GGCATCCAAGCAGCAAGC	Variable	[40]
3′-CS	R	AAGCAGACTTGACCTGAT		
Int1	F	AGCACCTTGCCGTAGAAGAACAG	3500	This study
	R	GTCATAATCGGTTATGGCATCGC		
intI1	F	GGGTCAAGGATCTGGATTTCG	1250	[33]
	R	ACATGCGTGTAAATCATCGTCG		
sul1	F	ATGGTGACGGTGTTCGGCATTCTGA	900	[34]
	R	TCTGGCTCCCAATCTAGTACGGATC		
qacEΔ1	F	ATCGCAATAGTTGGCGAAGT	600	[35]
	R	CAAGCTTTTGCCCATGAAGC		
aadA	F	ACATCATTCCGTGGCGTTATC	1100	This study
	R	TTATTTGCCGACTACCTTGGTGA		
tnpA	F	GGCGGGATCTGCTTGTAGAG	920	[36]
	R	CTCCGGAGATGTCTGGCTTACT		
aad B	F	TCACAGCCAAACTATCAGG	857	This study
	R	TGCTCCACCAATCACAAT		
aacA4	F	TGACCAACAGCAACGATTCC	800	[37]
	R	TTAGGCATCACTGCGTGTTC		
*bla*_OXA_	F	ATGAAAAACACAATACATATCAACTTCGC	900	This study
	R	GTGTGTTTAGAATGGTGATCGCATT		
*bla*_KPC_	F	TGTCACTGTATCGCCGTC	1000	[31]
	R	CTCAGTGCTCTACAGAAAACC		
*bla*_TEM_	F	CTGTCTATTTCGTTCATCC	1061	[24]
	R	CTCAGTATTGCCCGCTCC		
*bla*_VIM_	F	CACGAACCCAGTGGACATA	1512	This study
	R	TCACAGTAACCAGCAAATCA		
*bla*_IMP_	F	GCACGAACCCAGTTGACATA	1394	This study
	R	GTTTCTTCTTCCCACCATCC		
tniC	F	GCTCTGGTTGAGTTGGTG	993	This study
	R	GGATCCTTCCGCCTGTTG		
dfr	F	GTGAAAATATCACTAATGG	750	[38]
	R	TTAACCCCTTTGCCAGATTTG		
arr-3	F	GGTGACTTGCTAACCACAG	450	This study
	R	ACAGTGACATAGCAAGTTCAG		
catB	F	CCTGAAGATTGCCAAGAGTGGT	980	[39]
	R	AGTTTGTTCAGGGTGACGAAGG		

### Sequence annotation and genome comparison

ORFs were predicted with RAST (http://rast.nmpdr.org/) and further annotated using BLASTP and BLASTN programs (https://blast.ncbi.nlm.nih.gov/Blast.cgi) against UniProtKB/Swiss-Prot (http://web.expasy.org/docs/swiss-prot_guideline.html) and National Center for Biotechnology NR databases (https://www.ncbi.nlm.nih.gov/). Database annotation of drug-resistance genes, mobile elements and other genes was based on CARD (http://arpcard.mcmaster.ca), the β-lactamase (http://www.ncbi.nlm.nih.gov/pathogens/submit_beta_lactamase) database, ISfinder (https://www-is.biotoul.fr/), and INTEGRALL (http://integrall.bio.ua.pt/?). Sequence comparisons were performed with BLASTN and CLUSTALW2 (http://www.ebi.ac.uk/Tools/msa/clustalw2/). Gene organisation diagrams were drawn with Inkscape (https://inkscape. org).

### Carbapenemase activity detection

The activities of A, B and D carbapenemase classes in the bacterial cell extracts for the above mentioned six isolates were determined using a modified CarbaNP test [26]. Overnight bacterial cell cultures in Mueller-Hinton (MH) broth were each diluted 1:100 into 3 mL of fresh MH broth, and the bacteria were grown at 37 °C with shaking at 200 rpm to reach OD600s of 1.0 to 1.4. When required, ampicillin was used (200 μg/mL). Bacterial cells were harvested from 2 mL of each culture, and each pellet from the individual cultures was washed twice with 20 mM Tris-HCl (pH 7.8). Each cell pellet was resuspended in 500 μL of 20 mM Tris-HCl (pH 7.8), lysed by sonication and spun at 10000×g at 4 °C for 5 min. Each 50 μL supernatant (containing the enzymatic bacterial suspension fraction) was mixed with 50 μL of the following substrates (I to V), followed by incubation at 37 °C for a maximum of 2 h: substrate I: 0.054% phenol red plus 0.1 mM ZnSO4 (pH 7.8), substrate II: 0.054% phenol red plus 0.1 mM ZnSO4 (pH 7.8), and 0.6 mg/μL imipenem, substrate III: 0.054% phenol red plus 0.1 mM ZnSO4 (pH 7.8), 0.6 mg/μL imipenem, and 0.8 mg/μL tazobactam, substrate IV: 0.054% phenol red plus 0.1 mM ZnSO4 (pH 7.8), 0.6 mg/μL imipenem, and 3 mM EDTA (pH 7.8), and substrate V: 0.054% phenol red plus 0.1 mM ZnSO4 (pH 7.8), 0.6 mg/μL imipenem, 0.8 mg/μL tazobactam, and 3 mM EDTA (pH 7.8).

### Nucleotide sequence accession numbers

In*1021*_Nsa217_, In*843*_Ecl175_, In*846*_Eae634_, In*37*_Nsa243_, and In*62*_Kpn761_ sequences, along with In*1059*_Kp3349_, a novel sequence, were deposited in GenBank under the accession numbers KR338349, KR338350, KR338351, KR338352, KJ716225 and KM589496, respectively.

## Results and discussion

### Integron cloning experiments and antibiotic susceptibility testing

**Fig. 1:**
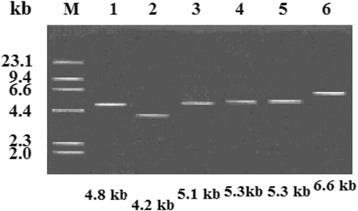
Electrophoretogram of recombinant plasmids encoding In1021, In843, In846, In37, In62, and novel In1059 with genetic mapping. *Lane M*, λ*Hind*III DNA marker (Amersham Biosciences, Shanghai, China). *Lane 1*, recombinant plasmids encoding In1021 from the isolate Nsa217 estimated to be approximately 4.8 kb-long; *lane 2*, recombinant plasmid encoding In843 from the isolate Ecl175 estimated to be approximately 4.1 kb-long; *lane 3*, recombinant plasmid encoding In846 from the isolate Eae634 estimated to be approximately 5.1 kb-long; *lane 4*, recombinant plasmid encoding In37 from the isolate Nsa243 estimated to be approximately 5.3 kb-long; *lane 5*, recombinant plasmid encoding In62 from the isolate Kpn761 estimated to be approximately 5.3 kb-long; *lane 6*, recombinant plasmid encoding novel In1059 from the isolate Kp3349 estimated to be approximately 6.6 kb-long

Six of 69 isolates, which resistance to aminoglycoside, quinolone, cephalosporin, and carbapenem antibiotics, from the clinical patients met the requirements of this study. Isolates Kpn761, Eae634, and Kp3349 were collected from blood culture, head wound secretion, and sputum, respectively, from ICU patients. Isolate Ecl175 came from a blood culture from a respiratory medical patient. Isolates Nsa217 and Nsa243 were collected from patient blood cultures in the hospital’s Infectious Disease Unit. Following BamHI digestion and ligation to a pMD19-T cloning vector, the recombinant plasmids were transformed into the competent cells, *E. coli* TOP10, by heat shock conversion, then, achieved the positive transformants which were selected by blue-white spot experiments, and sequenced. The six integron-containing recombinant plasmids transformed into *E. coli* TOP10 cells were used for the integron cloning conjugation experiments. The susceptibility test results are listed in Table 2. Concurrently, the six recombinant plasmids were electrophoresed to estimate their sizes (Fig. 1). The susceptibility test results indicated that the conjugation experiments were successful and that the resultant antibiotic resistance was caused by plasmid-mediated genes. The electrophoresis results following BamHI digestion indicated that the sizes of In1021, In843, In846, In37, In62 and the novel In1059 integron were ~4.8 kb, 4.2 kb, 5.1 kb, 5.3 kb, 5.3 kb and 6.6 kb each in length, respectively (Fig. 1). Usually, class 1 integrons that integrate with transposons or are encoded on plasmids can express regular mobilization and transformation capabilities, but they lack self-mobility [5, 13]. In view of these features, integrons can change from one type to another, with the possibility of generating novel types (Fig. 3).

**Table 2:** Antimicrobial drug susceptibility profiles

Strains	Plasmids	Antibiotic susceptibility testing (mg/L)											
		Cephalosporins			Carbapenems			Aminoglycosides			Fluoroquinolones		
		CZ	CAZ	CTX	ETM	MPM	IPM	NET	TOB	AK	NOR	OFL	CIP
Nsa243	In*37*	256/R	128/R	128/R	16/R	16/R	8/R	64/R	32/R	128/R	0.10/S	0.05/S	0.25/S
	In*37*-TOP10	16/R	8/R	8/R	8/R	8/R	4/R	16/R	16/R	32/R	0.05/S	0.003/S	0.125/S
	TOP10	1/S	0.5/S	0.5/S	0.5/S	0.5/S	0.25/S	2/S	0.025/S	1/S	0.05/S	0.003/S	0.125/S
Ecl175	In*843*	2/S	1/S	1/S	0.5/S	0.5/S	0.25/S	512/R	128/R	512/R	0.10/S	0.05/S	0.25/S
	In*843*-TOP10	1/S	0.5/S	0.5/S	0.25/S	0.25/S	0.125/S	128/R	64/R	128/R	0.05/S	0.003/S	0.125/S
	TOP10	1/S	0.5/S	0.5/S	0.5/S	0.5/S	0.25/S	64/R	32/R	64/R	0.05/S	0.003/S	0.125/S
Kpn761	In*62*	2/S	1/S	1/S	0.5/S	0.5/S	0.25/S	128/R	64/R	128/R	0.10/S	0.05/S	0.25/S
	In*62*-TOP10	1/S	0.5/S	0.5/S	0.25/S	0.25/S	0.125/S	64/R	32/R	32/R	0.05/S	0.003/S	0.125/S
	TOP10	1/S	0.5/S	0.5/S	0.5/S	0.5/S	0.25/S	2/S	0.025/S	1/S	0.05/S	0.003/S	0.125/S
Nsa217	In*1021*	2/S	1/S	1/S	0.5/S	0.5/S	0.25/S	256/R	128/R	256/R	0.10/S	0.05/S	0.25/S
	In*1021*-TOP10	1/S	0.5/S	0.5/S	0.25/S	0.25/S	0.125/S	128/R	64/R	128/R	0.05/S	0.003/S	0.125/S
	TOP10	1/S	0.5/S	0.5/S	0.5/S	0.5/S	0.25/S	2/S	0.025/S	1/S	0.05/S	0.003/S	0.125/S
Eae634	In*846*	256/R	128/R	128/R	64/R	64/R	64/R	128/R	64/R	128/R	0.10/S	0.05/S	0.25/S
	In*846*-TOP10	64/R	16/R	16/R	16/R	16/R	16/R	64/R	32/R	64/R	0.05/S	0.003/S	0.125/S
	TOP10	1/S	0.5/S	0.5/S	0.5/S	0.5/S	0.25/S	2/S	0.025/S	1/S	0.05/S	0.003/S	0.125/S
KP3349	In*1059*	256/R	128/R	128/R	64/R	64/R	128/R	64/R	128/R	64/R	0.10/S	0.05/S	0.25/S
	In*1059*-TOP10	32/R	16/R	16/R	16/R	16/R	64/R	32/R	16/R	16/R	0.05/S	0.003/S	0.125/S
	TOP10	1/S	0.5/S	0.5/S	0.5/S	0.5/S	0.25/S	2/S	0.025/S	1/S	0.05/S	0.003/S	0.125/S

*Abbreviations*: *CZ* Cefazolin, *CAZ* Ceftazidime, *EPM* Ertapenem, *MPM* Meropenem, *IPM* Imipenem, *NET* Netilmicin, *TOB* Tobramycin, *AK* Amikacin, *NOR* Norfloxacin, *OFL* Ofloxacin, *CIP* Ciprofloxacin

### Strain carbapenemase activities and genetic features of integrons

The following strains (transformants) In37-TOP10, In846-TOP10 and In1059-TOP10 have class D, B and B carbapenemase activities, respectively, while In37 (isolate Nsa243) appears to have class D activity, while In846 (Eae634) and In1059 (Kp3349) appear to have class B activities, respectively (data not shown). All the above strains were resistant to the cephalosporin, carbapenem and aminoglycoside drugs but they were susceptible to fluoroquinolones (Table 2). In62 apparently represents the most primitive form among these integrons. In62 carries two different resistance markers, while In37, In843, In846, In1021 and In1059 have evolved to capture the determinants of at least three different antibiotic classes, and they most likely confer MDR.

**Fig. 2:**
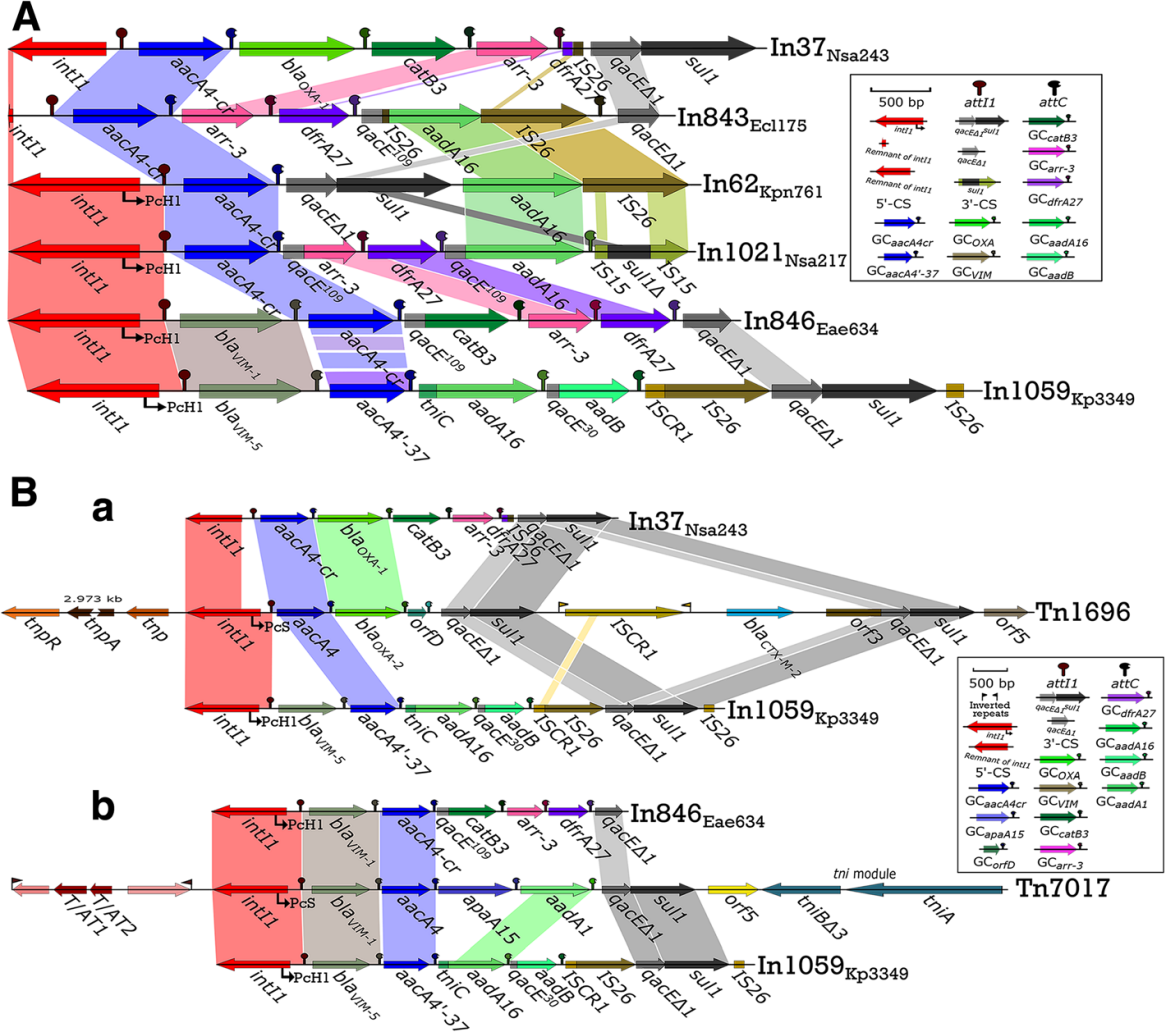
Integron comparisons based on BamHI digestion of plasmids. Genes are denoted by *arrows* and *colours* based on the gene function classifications. *Shaded areas* denote regions with >99% nucleotide sequence identity, with the exception of >97% for the comparison of GC*aacA4cr* in In846 and GC*aacA4′-37* in novel In1059. **A**, Comparison of In37, In843, In62, In1021, In846 and novel In1059 integrons after BamHI digestion. **B**-*a*, Comparison of integrons In37, novel In1059 and transposon Tn1696 (DQ310703); **B**-*b*, Comparison of integrons In846, novel In1059 and transposon Tn7017 (KJ571202). The novel In1059 integron shares higher sequence identity with integron Tn7017 than with integron Tn1696

**Fig. 3:**
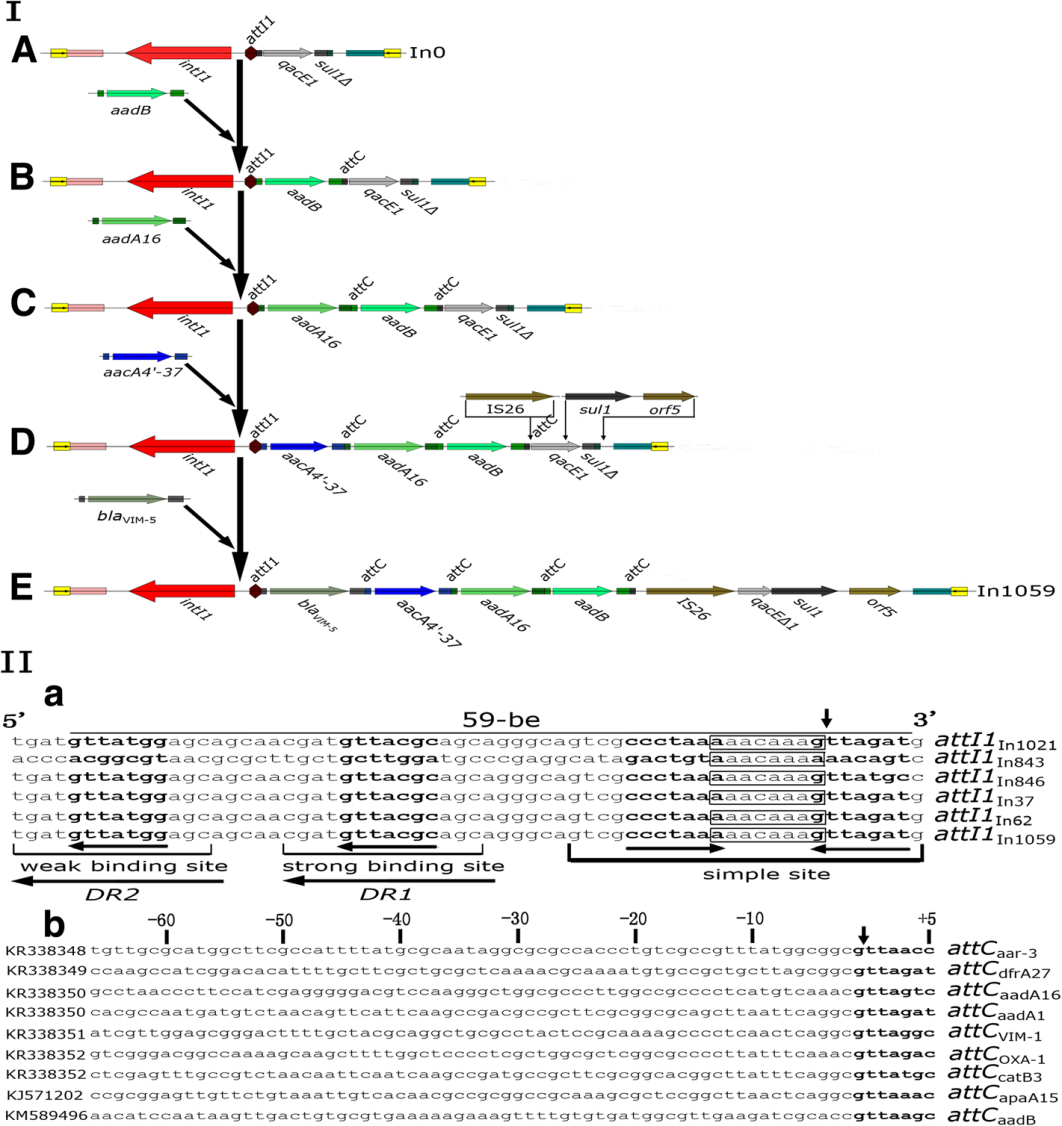
Genetic structures and proposed mobilization and evolution of In0 to In1059 and the analysis of *intI1* and *attC* in class 1 integrons from this study. I, genetic structures and putative mobilization. A, In0; B, A + GC*aadB*; C, B + GC*aadA16*; D, C + GC*aacA4’-3*; E, In1059 = D + GC*bla*_VIM-5_ *+* IS26 + (*sul1* + *orf5*) (replacing *sulΔ1*); II, Sequences and structures of *attI1* (59-be, 59-base elements) and *attC.* II-a, the *attI1* (59-be) site positions are denoted by the *bold black line*. *Open bars* indicate potential *IntI1* recognition sites, and *arrows* under the 7-bp core sites indicate relative orientations. The 7-bp core site sequences are shown in *bold type*, and the strong and weak *IntI1*-binding sites and direct repeats, *DR1* and *DR2*, are indicated in accordance with the literature [29]. The simple site locations are denoted by an *open bar*. The “*aacaaag(a)*” sequences in the *black boxes* are reminiscent of the spacer in *attI1* and occur between the two gene cassettes; recombination or excision of gene cassettes occurs frequently at these sites as indicated in *panel I*, process C to D. *II-b*, the *attC* sequences of class 1 integrons. The *bold black* letters denote the 7-bp core site, while the numbers are the nucleotide positions (*left, negative, right, positive*) of the recombination *cross-over points*, as indicated by a *vertical arrow* where the recombination or excision of gene cassettes regularly occurs, as shown in *panel I*. The GenBank accession numbers are shown on the *left-hand side* of the diagram

In1059 and In846 integrons differed markedly from the other integrons (In37, In843, In62 and In1021) in this study (Table 3 and Additional file 1). Seemingly, their genetic differences appeared to involve the presence of an additional gene cassette containing *bla*_VIM_ downstream of the *intI1* gene when compared with the other integrons in this study (Fig. 2a). Therefore, the biochemistries of In1059 and In846 should differ from the other integrons. Four gene cassettes and one remnant of *dfrA27* were identified in In*37* between *aacA4cr* gene cassette (GC*aacA4cr*) and GC*catB3*, while the existence of GC*bla*_OXA-1_ indicates that this integron differs in its biochemistry to In843, In62 and In1021. In37 and In843 differ from In62, In846, In1021 and In1059 class 1 integrons in their 5′-CSs (remnant of *intI1*), and both lack Pc variant (Table 3 and Additional file 1). In62 appears to have the basic pattern identified in the six class 1 integrons, all of which commonly contain GC*aacA4cr*. But only GC*aacA4cr* exists in In62. Notably, one *aadA16* and four gene cassetts in the In843 gene are inserted into IS26 in two parts, before reintegrating with the *qacE* ^*109*^ (109 nucleotide remanants of *qacE*) gene (Table 3 and Additional file 1). Unusually, some fragments such as *qacE* ^*109*^ or *qacE* ^*30*^ combined with GC*aadA16* in In843 and In1021, GC*arr-3* in In1021 and In846, and GC*aadB* in In1059 have never been recovered from any class 1 integron to date, and these may have “pseudo-gene cassette” functions (Table 3 and Additional file 1). Indeed, *qacE* ^*101*^ was discovered in class 1 integrons [27], and no *qacE* ^*30*^ or *qacE* ^*109*^ elements have previously been found. In the novel In1059 integron, the GC*aacA4* gene coding sequence displays a previously unseen duplication and an unusual 9 bp deletion (cccttcCAT) in its *attC*, thereby inactivating it (Table 3 and Additional file 1). The duplication extends the sequence to 576-bp in length instead of the usual 555-bp. The resulting gene, which is a novel allele, has been assigned aac*A4’-37* (Fig. 3 and Table 3 and Additional file 1), and the fragment itself appears to be embedded within the start of GC*aadA16*, resulting in a change in its right-hand part of *attC* site (e.g. the motif locates upstream of the coding sequence), and the remaining part of the *attC* site in GC*aadA16* seems to be a hybrid between the usual one in GC*aadA16* and the usual one in GC*aacA4* (*attC*_aadA16/aacA4_, Table 3 and Additional file 1); furthermore, the GC*aadB* sequence lacks a right-hand *attC* part making it look like a pseudo-gene cassette (Table 3 and Additional file 1). However, GC*bla*_VIM-5_ appears to be usual *attC* and normal gene cassette functional in novel In1059 integron.

**Table 3:** Comparison of novel In1059 with other integrons in the study

Integron	In1059	In846	In843	In37	In62	In1021
Pc variant	PcH1	PcH1	None	None	PcH1	PcH1
	“-35”: tggaca	“-35”: tggaca			“-35”: tggaca	“-35”: ggacat
	“-35”: taaact	“-10”: taaact			“-10”: ttcgta	“-10”: taaact
P2 promoter	Absent	Absent	Absent	Absent	Absent	Absent
PintI1	Yes	Yes	Yes	Yes	Yes	Yes
	“-10”: agtcta	“-10”: agtcta	“-10”: agggcg	“-10”: agtcta	“-10”: agtcta	“-10”: agtcta
	“-35”: cagcaa	“-35”: cagcaa	“-35”: catcgt	“-35”: cagcaa	“-35”: cagca	“-35”: cagcaa
19 bp ORF11 duplication	No	No	No	No	No	No
*intI1*	*IntI1*_R32_H39_	*IntI1*_R32_H39_	Remant of *intI1*	Remant of *intI1*	*IntI1*_R32_H39_	*IntI1*_R32_H39_
	1010 bp-length	1014 bp-length	4 bp-length	720 bp-length	1014 bp-length	1014 bp-length
*attI1*	63 bp-length	63 bp-length	63 bp-length	63 bp-length	63 bp-length	63 bp-length
Array of gene cassettes	GC*bla*_VIM-5_ - GC*aacA4’-37*- (*tniC*-*aadA16*)- (*qacE* ^*30*^-*aadB*)	GC*bla*_VIM-1_ - GC*aacA4cr*- (*qacE* ^101^-*catB3*) - GC*arr-3*	GC*aacA4cr* - GC*arr-3* - GC*dfrA27* - (*qacE* ^101^-*aadA16Δ*::IS26)	GC*aacA4cr* - GC*bla*_OXA-1_ - GC*catB3* - GC*arr-3*	GC*aacA4cr*	GC*aacA4cr* - (*qacE* ^*109*^-*arr-3*) - *GCdfrA27* - (*qacE* ^*30*^-*aadA16*)
*attC*	*attCbla*_VIM-5_(L;R) *attC*_aacA4Δ_(LΔ; R); *attC*_aadA16/aacA4_(L;R); *attC*_aadBΔ_(L; Del)	*attCbla*_VIM-1_(L;R); *attC*_aacA4cr_(L;R); *attC*_catB3_ (L;R); *attC*_aacA4_(LΔ; R)	*attC*_aacA4cr_ (L; R); *attC*_arr-3_ (L;R); *attC*_dfrA27_ (L;R); *attC*_aadA16_ (L;R)	*attC*_aacA4cr_ (L;R); *attCbla*_OXA-1_(L;R); *attC*_catB3_ (L;R); *attC*_arr-3_ (L; R)	*attC*_aacA4cr_ (L; R)	*attC*_aacA4cr_ (L;R); *attC*_arr-3_ (L; R); *attC*_dfrA27_ (L; R); *attC*_aadA16_(L;R) mutated
3′-CS	*qacEΔ1-sul1*	*qacEΔ1* (partial)	*qacEΔ1* (partial)	*qacEΔ1-sul1*	*qacEΔ1-sul1*	IS15-*sul1Δ*-IS1

*Abbreviations: attC (L; R)* left- and right-hand parts of *attC* in gene cassettes, *attC (LΔ; R)* truncated left- and normal right- hand parts of *attC* in gene cassettes, *attC (L; Del)* normal left- and deleted right-hand parts of *attC* in gene cassettes, suggesting no functional gene cassette, *attC (L; R) mutated* mutated left- and mutated right-hand parts of *attC* in gene cassettes, *GC* abbreviation of gene cassette

Sequence comparisons of InV117 [28] (part of Tn1696, GenBank accession number DQ310703), In1059 and In37 reveal high levels of sequence homology among them (Fig. 2B-a), and In37 in particular shares high sequence identity with In1059*.* Because the Tn1696 module (including InV117) has the potential to transpose, this suggests that In37 and In1059 should also potentially be mobile elements. Compared with In70 [20] (part of Tn7017, GenBank accession number KJ571202), In1059 and In843 share the most sequence homology with In70 (Fig. 2B-b), and this is especially so for novel In1059*.* These results provide new insight about the structure of In1059, which coupled with the known plasticity of the In70 genetic context in the recombination plasmids, means that this might have mediated mobilization of the *bla*_VIM-1_-containing In70 integron platform [21].

### Mobilization and evolutionary inferences for In0 to In1059

Generally, In0 could emerge from excision of all cassettes of an integron. If In0 captures GC*aadB* on *attI1* site likely under antibiotic pressure, structure A might be transferred to structure B, maybe becoming a novel integron (Fig. 3I); then, likely under similar conditions, if joining with GC*aadA16*, structure B might become structure C, too (Fig. 3I); Also, if joined by GC*aacA4’-3*, structure C might turn into structure D, perhaps involving a novel integron as well; meanwhile, if inserted into GC*bla*_VIM-5_ on *attI1* site and IS26 on site between GC*aadB* and *qacE1*, then, replaced *sul1Δ* with *sul1* and *orf5,* structure D will convert into structure E, likely under the right conditions, just as novel In1059 assigned in the study (Fig. 3 and Table 3 and Additional file 1). According to Collis et al., 1998 [29], the recombination or excision of gene cassettes from class 1 integrons frequently occurs at sites of vertical arrow (Fig. 3II-a) between the ″*aacaaag(a)*″ array and its next nucleotide in *attI1*, and sites of vertical arrow (Fig. 3II-b) between ″*g*″ and ″*t*″ of ″*gtttrrry*″ array in *attC*; however, the study appears slightly mutant sequences in terms of the cross-over point of *attI1* and *attC* (Fig. 3II-a and b). Integrons are associated with MDR in Gram-negative bacteria [30, 31, 32] and have been determined as the primary source of drug-resistance genes, and they are also suspected to be reservoirs of drug-resistance genes in patients with bacterial infections [20].

## Conclusions

Antibiotic overuse makes it likely that more and more MDR strains will appear in clinical isolates, and most of these strains will contain class 1 integrons. The features of the integrons described above denote a scheme involving the stepwise and parallel evolution of class 1 integron variation likely under antibiotic selection pressure in clinical settings.

## Additional files

Additional file 1: Analysis for integrons: In1059, In843, In846, In37 and In62. (XLS 48 kb)
